# Experimental Validation of Entropy-Driven Swarm Exploration under Sparsity Constraints with Sparse Bayesian Learning

**DOI:** 10.3390/e24050580

**Published:** 2022-04-20

**Authors:** Christoph Manss, Isabel Kuehner, Dmitriy Shutin

**Affiliations:** 1 German Research Center on Artifical Intelligence, Marie-Curie-Straße 1, 26129 Oldenburg, Germany; 2 German Aerospace Center, Münchener Straße 22, 82234 Wessling, Germany; isabel.kuehner@dlr.de (I.K.); dmitriy.shutin@dlr.de (D.S.)

**Keywords:** distributed estimation, Sparse Bayesian Learning, exploration, swarm, multi-agent systems, consensus, D-optimal design

## Abstract

Increasing the autonomy of multi-agent systems or swarms for exploration missions requires tools for efficient information gathering. This work studies this problem from theoretical and experimental perspectives and evaluates an exploration system for multiple ground robots that cooperatively explore a stationary spatial process. For the distributed model, two conceptually different distribution paradigms are considered. The exploration is based on fusing distributively gathered information using Sparse Bayesian Learning (SBL), which permits representing the spatial process in a compressed manner and thus reduces the model complexity and communication load required for the exploration. An entropy-based exploration criterion is formulated to guide the agents. This criterion uses an estimation of a covariance matrix of the model parameters, which is then quantitatively characterized using a D-optimality criterion. The new sampling locations for the agents are then selected to minimize this criterion. To this end, a distributed optimization of the D-optimality criterion is derived. The proposed entropy-driven exploration is then presented from a system perspective and validated in laboratory experiments with two ground robots. The experiments show that SBL together with the distributed entropy-driven exploration is real-time capable and leads to a better performance with respect to time and accuracy compared with similar state-of-the-art algorithms.

## 1. Introduction

For exploration tasks that rely on multi-agent systems, with complex, unstructured terrains, autonomy plays a key role to lower potential threats or tedious work for human operators, be it space exploration, disaster relief, or routine industrial facility inspections. The main objective here is to give a human operator more detailed information about the explored area, e.g., in terms of a map, and to support further decision making. While multiple agents do provide an increased sensing aperture and can potentially collect information more efficiently than a single-agent system, they have to rely more heavily on autonomy to compensate, e.g., possible large (or unreliable) communication delays [1] or the complexity of teleoperating multiple agents.

One of the approaches to increase the autonomy of multi-agent systems consists of using in situ analysis of the collected data with the agents’ own computing resources to decide on future actions. In the context of mapping, such an approach is also known as active information gathering [2,3] or exploration. Note that mapping is generally not restricted to sensing with imaging sensors, such as cameras. The exploration of gas sources [4] or of the magnetic field [5] also falls in this category.

An approach for active information gathering lies in the focus of the presented work. In the following, we provide an overview of work related to the approach discussed in this paper, the arising challenges, and a proposed solution.

### 1.1. Related Work

The objective of active information gathering is to utilize the collected data, represented in terms of a parameterized model, to compute information content as a function of space. This can be done using heuristic approaches, as in [6,7], where the authors modify the random walk strategy by adjusting the movement steps of each robot such as to collect more information. Alternatively, information–theoretic approaches can be used. In [8], the authors use a probabilistic description of the model to steer cameras mounted on multiple unmanned aerial vehicles (UAVs). In this case, the information metric can be computed directly based on statistics of the pixels. The resulting quantity is then used to autonomously coordinate UAVs in an optimal configuration. In [9], the authors propose an *exploration driven by uncertainty* by minimizing the determinant of the covariance matrix for an optimal camera placement for a 3D image. This approach essentially implements an optimal experiment design [10], which in turn relates the determinant of the covariance matrixof the model parameters to the Shannon entropy of Gaussian random variables. This connection has been further explored in [11], where the authors compare criteria for optimal experiment design with mutual information for Gaussian processes regression and sensor placement. This leads to a greedy algorithm that uses mutual information for finding optimal sensor placements. An extension of [11] for multiple agents and a decentralized estimation of the mutual information is presented in [2,12]. In the latter, the authors also considered robotic aspects, such as optimal trajectory planning along with information gathering: an approach that has been further investigated in [13].

One of the key elements in experiment design-based information gathering is the ability to compute the covariance structure of the model parameters as a function of space and evaluate it in a distributed fashion. In [14], the authors studied the information-gathering approach for sparsity constrained models, i.e., under assumption that the model parameters are sparse. This required implementing non-smooth $\ell_{1}$ constraints in the optimization problem, which in turn made the exact computation of the parameter covariance impossible. Instead, the covariance structure was approximated by locally smoothing the curvature of the objective function. In [14], the method was applied to generalized linear models with sparsity constraints for a distributed computation with two versions of data splitting over agents: homogeneous splitting, also called splitting-over-examples (SOE), and heterogeneous splitting, also called splitting-over-features (SOF). However, despite the method yielding in simulations a better performance as compared to systematic or random exploration approaches, the used approximation has been derived with purely empirical arguments.

### 1.2. Paper Contribution

To address this, the exploration problem with sparsity constraints has been cast into a probabilistic framework, where the parameter covariance can be computed exactly. In [15], we formulated a Bayesian approach toward cooperative sparse parameter estimation for SOF, and in [16] for SOE data splitting. However, the distributed computation of the covariance matrix and information-driven exploration has not been considered so far. With this contribution, we close this gap and study an information-driven exploration strategy that is based on a Bayesian approach toward distributed sparse regression. Specifically,  

- We consider a distributed computation of the corresponding parameter covariance matrices for information-seeking exploration using a Bayesian formulation of the model, and
- Validate the algorithm’s performance both in simulations as well as in an experiment with two robots exploring the magnetic field variations on a laboratory floor.  

The rest of the paper is structured as follows. We begin with a model formulation and model learning in Section 2. In Section 3, we discuss a distributed computation of the exploration criterion for the considered regression problem. Afterwards, we outline the experimental setting, the collection of ground truth data, and the sensor calibration in Section 4, as well as the overall system design in Section 5. The experimental results are summarized in Section 6, and Section 7 concludes this work.

## 2. Distributed Sparse Bayesian Learning

### 2.1. Model Definition

We make use of a classical basis function regression [17] to express an unknown scalar physical process p(x)∈R, with $x\in R^{d}$ and d∈N. Typically, the process is *d*-dimensional, with d∈{2,3}. To represent the process p(x), a set of N∈N basis functions $\phi_{n}\left(x,\pi_{n}\right)\in R$, n=1,…,N are used, where $\pi_{n}\in R^{s}$ is dependent on the used basis function and *s* is a number of parameters per basis function.

Each basis function is parameterized with $\pi_{n}$, n=1,…,N, which can represent centers of corresponding basis functions, their width, etc. More formally, we assume that

(1) $$\begin{array}{c} p\left(x\right)=\sum_{n=1}^{N}\phi_{n}\left(x,\pi_{n}\right)w_{n}, \end{array}$$

where $w_{n}\in R$ are generally unknown weights in the representation.

To estimate $w_{n}$, n=1,…,N, we make *M* observations of the process p(x) at locations $X={\left[x_{1},\cdots ,x_{M}\right]}^{T}\in R^{M\times d}$. The corresponding *m*-th measurement is then represented as

(2) $$\begin{array}{c} y\left(x_{m}\right)=p\left(x_{m}\right)+\eta \left(x_{m}\right)=\sum_{n=1}^{N}\phi_{n}\left(x_{m},\pi_{n}\right)w_{n}+\eta \left(x_{m}\right), \end{array}$$

where $\eta \left(x_{m}\right)\propto N\left(0,\lambda^{-1}\right)$ is an additive sample of white Gaussian noise with a known precision $\lambda \in R_{+}$. By collecting *M* measurements in a vector $y\left(X\right)={\left[y\left(x_{1}\right),\cdots ,y\left(x_{M}\right)\right]}^{T}$$\in R^{M}$, we can reformulate (2) in a vector-matrix notation. To this end, we define

(3) $$\begin{array}{cc} \Pi & ≜{\left[\pi_{1},\ldots ,\pi_{N}\right]}^{T}\in R^{N\times s}, \end{array}$$

(4) $$\begin{array}{cc} \phi_{n}\left(X,\pi_{n}\right) & ≜{\left[\phi_{n}\left(x_{1},\pi_{n}\right),\cdots ,\phi_{n}\left(x_{M},\pi_{n}\right)\right]}^{T}\in R^{M}, \end{array}$$

(5) $$\begin{array}{cc} \Phi (X,\Pi ) & ≜\left[\phi_{1}\left(X,\pi_{1}\right),\cdots ,\phi_{N}\left(X,\pi_{N}\right)\right]\in R^{M\times N}, \end{array}$$

(6) $$\begin{array}{cc} \mathrm{and}\;\;\;w & ≜{\left[w_{1},\cdots ,w_{N}\right]}^{T}\in R^{N}, \end{array}$$

which allows us to formulate the measurement model in a vectorized form

(7) $$\begin{array}{c} y(X)=\Phi (X,\Pi )w+\eta (X), \end{array}$$

with $\eta \left(X\right)≜{\left[\eta \left(x_{1}\right),\cdots ,\eta \left(x_{M}\right)\right]}^{T}\in R^{M}$.

Based on (7), we define the likelihood of the parameters w as follows

(8) $$p\left(y\left(X\right)|w\right)\propto \exp\left\{-\frac{\lambda }{2}{∥y\left(X\right)-\Phi \left(X,\Pi \right)w∥}^{2}\right\}.$$

Often, the representation (1) is selected such that N≫M, i.e., it is underdetermined. This implies that there is an infinite number of possible solutions for w. A popular approach to restrict a set of solutions consists of introducing sparsity constraints on parameters. Within the Bayesian framework, this can be achieved by defining a prior over the parameter weights w. This leads to a class of probabilistic approaches referred to as Sparse Bayesian Learning (SBL).

The basic idea of SBL is to assign an appropriate prior to the *N*-dimensional vector w such that the resulting maximum a posteriori (MAP) estimate $\hat{w}$ is sparse, i.e., many of its entries are zero. Typically, SBL specifies a hierarchical factorable prior $p\left(w|\gamma \right)p\left(\gamma \right)=\prod_{n=1}^{N}p\left(w_{n}|\gamma_{n}\right)p\left(\gamma_{n}\right)$, where $p\left(w_{n}|\gamma_{n}\right)=N\left(w_{n}|0,\gamma_{n}\right)$, n∈{1,…,N} [18,19,20]. For each n∈{1,…,N}, the hyperparameter $\gamma_{n}$, also called sparsity parameter, regulates the width of $p(w_{n}|\gamma_{n})$; the product $p\left(w_{n}|\gamma_{n}\right)p\left(\gamma_{n}\right)$ defines a Gaussian scale mixture (the authors in work [21] extend this framework by generalizing $p(w_{n}|\gamma_{n})$ to be the probability density function (PDF) of a power exponential distribution, which makes the hierarchical prior a power exponential scale mixture distribution). Bayesian inference on a linear model with such a hierarchical prior is commonly realized via two types of techniques: MAP estimation of w (Type I estimation; note that many traditional “non-Bayesian” methods for learning sparse representations such as basis pursuit de-noising or re-weighted $\ell_{p}$-norm regressions [22,23,24] can be interpreted as Type I estimation within the above Bayesian framework [21]) or MAP estimation of γ (Type II estimation, also called maximum evidence estimation, or empirical Bayes method). Type II estimation has proven (both theoretically and empirically) to perform consistently better than Type I estimation in the present application context. One reason is that the objective function of a Type II estimator typically exhibits significantly fewer local minima than that of the corresponding Type I estimator and promotes greater sparsity [25]. The hyperprior $p(\gamma_{n})$, n∈{1,…,N}, is usually selected to be non-informative, i.e., $p\left(\gamma_{n}\right)\propto \gamma_{n}^{-1}$ [26,27,28]. The motivation for this choice is twofold. First, the resulting inference schemes typically demonstrate superior (or similar) performance as compared to schemes derived based on other hyperprior selections [21]. Second, very efficient inference algorithms can be constructed and studied [26,27,28,29,30].

In the following, we consider only SBL Type II optimization as it leads to usually sparser parameter vectors w [21], and we drop explicit dependencies on measurements X and basis function parameters Π to simplify notation. The marginalized likelihood for SBL Type II optimization is therefore

(9) $$\begin{array}{c} p\left(y|\gamma \right)=\int_{-\infty }^{\infty }p\left(y|w\right)p\left(w|\gamma \right)dw\propto {\left|\Sigma \right|}^{-\frac{1}{2}}\exp\left\{-\frac{1}{2}y^{T}\Sigma^{-1}y\right\}, \end{array}$$

where $\Sigma =\lambda^{-1}I+\Phi \Gamma \Phi^{T}$, Γ=diag{γ}, and I being the identity. Taking the negative logarithm of (9), we obtain the objective function for SBL Type II optimization in the following form

(10) $$\begin{array}{c} L\left(\gamma \right)=-\log p\left(y|\gamma \right)=\log\left(|\Sigma |\right)+y^{T}\Sigma^{-1}y. \end{array}$$

An estimate of hyperparameters γ is then found as

(11) γ^=arg min γL(γ).

Once the estimate $\hat{\gamma }$ is obtained, the posterior probability density function (PDF) of the the parameter weights w can be easily computed: it is known to be Gaussian $p\left(w|y,\hat{\gamma }\right)=N\left(\hat{w},\Sigma_{w}\right)$ with the moments given as

(12) $$\begin{array}{ccc} \hat{w}=\lambda \Sigma_{w}\Phi^{T}y, & \; & \Sigma_{w}={\left(\lambda \Phi^{T}\Phi +{\hat{\Gamma }}^{-1}\right)}^{-1}, \end{array}$$

where $\hat{\Gamma }=\mathrm{diag}\left\{\hat{\gamma }\right\}$ (see also [18]).

### 2.2. Sparse Bayesian Learning with the Automatic Relevance Determination

The key to a sparse estimate of w is a solution to (11). There are a number of efficient schemes [26,27,28] to solve this problem. The method that we use in this paper is based on [26]. In the following, we shortly outline this algorithm.

In [26], the authors introduced the reformulated automatic relevance determination (R-ARD) by using an auxiliary function that upper bounds the objective function L(γ) in (10). Specifically, using the concavity of the log-determinant in (10) with respect to γ, the former can be represented using a Fenchel conjugate as

(13) $$\begin{array}{c} \log\left|\Sigma \right|=\min_{z}\;z^{T}\gamma -h^{*}\left(z\right), \end{array}$$

where $z\in R^{N}$ is a dual variable and $h^{*}\left(z\right)$ is the dual (or conjugate) function (see also [31] (Chapter 5) or [32]).

Using (13), we can now upper-bound (10) as follows

(14) $$\begin{array}{c} L\left(\gamma ,z\right)≜z^{T}\gamma -h^{*}\left(z\right)+y^{T}\Sigma^{-1}y\geq L\left(\gamma \right). \end{array}$$

Note that for any γ, the bound becomes tight when minimized over z. This fact is utilized for the numerical estimation of γ, which is the essence of the R-ARD algorithm.

R-ARD alternates between estimating z, which can be found in closed form as [26,31]

(15) z^=arg min zL(γ^,z)=∂∂γlog|Σ|γ=γ^=diag{ΦTΣ−1Φ},

and estimating $\hat{\gamma }$ as a solution to a convex optimization problem

(16) γ^=arg min γL(γ,z^)=arg min γz^Tγ+yTΣ−1y.

In order to solve (16), the authors in [26] proposed to use yet another upper bound on L(γ,z). Specifically, by noting that

(17) $$\begin{array}{c} y^{T}\Sigma^{-1}y=\min_{w}\;\lambda {∥y-\Phi w∥}^{2}+\sum_{l=1}^{N}\frac{w_{l}^{2}}{\gamma_{l}} \end{array}$$

the cost function in (16) can be bounded with

(18) L(w,γ,z^)≜λ∥y−Φw∥2+∑l=1Nz^lγl+wl2γl≥L(γ,z^).

The right-hand side of (18) is convex both in w and γ. As such, for any fixed w, the optimal solution for γ can be easily found as γl=z^l−12|wl|, l=1,…,N. By inserting the latter in (18), we find the solution for w that minimizes the upper-bound $L(w,\gamma ,\hat{z})$ as

(19) w^=arg min wL(w,γ^,z^)=arg min wλ∥y−Φw∥2+2∑l=1Nz^l12|wl|,

which can be recognized as a weighted least absolute shrinkage and selection operator (LASSO) cost function. Expression (19) builds a basis for a distributed estimation learning of SBL parameters, since there exist techniques to optimize a LASSO function over a network, which are presented in the following section.

### 2.3. The Distributed Automated Relevance Determination Algorithm for SOF Data Splitting

The derivation of the distributed R-ARD (D-R-ARD) for SOF is shown in [14]. Here, we would like to show the main aspects of the distribution paradigm and the resulting algorithm. The main aspect of heterogeneous data splitting is that each agent has its own model. Therefore, the parameter weights w are distributed among K∈N agents as $w={\left[w_{1}^{T},\cdots ,w_{K}^{T}\right]}^{T}$ and each agent has its part $w_{k}\in R^{N_{k}}$, where $N=\sum_{k=1}^{K}N_{k}$. Likewise, the matrix Φ is partitioned among *K* agents as $\Phi =[\Phi_{1},\ldots ,\Phi_{K}]$ where $\Phi_{k}\in R^{M\times N_{k}}$. The SOF model is then formulated as

(20) $$\begin{array}{c} y=\left[\begin{array}{ccc} \Phi_{1} & \cdots & \Phi_{K} \end{array}\right]\left[\begin{array}{c} w_{1}\\ \vdots \\ w_{K} \end{array}\right]+\eta =\sum_{k=1}^{K}\Phi_{k}w_{k}+\eta . \end{array}$$

Similarly, the hyper-parameters γ are also partitioned as $\gamma ={\left[\gamma_{1}^{T},\cdots ,\gamma_{K}^{T}\right]}^{T}$.

The solution to cooperative SOF inference then amounts to computing z from (15) and optimizing the upper bound (18) over a network of *K* agents.

Unfortunately, in the case of the SOF model, the dual variable $z={\left[z_{1}^{T},\cdots ,z_{K}^{T}\right]}^{T}$ in (15) cannot be computed exactly. Instead it is upper-bounded [14] as $z_{k}\leq {\tilde{z}}_{k}$, where ${\tilde{z}}_{k}$ is computed for each agent:

(21) $$\begin{array}{c} {\tilde{z}}_{k}=\mathrm{diag}\left\{\Phi_{k}^{T}\Lambda \Phi_{k}-\Phi_{k}^{T}\Lambda \Phi_{k}\Sigma_{w,k}\Phi_{k}^{T}\Lambda \Phi_{k}\right\}, \end{array}$$

with $\Sigma_{w,k}={\left(\Phi_{k}^{T}\Lambda \Phi_{k}+\Gamma_{k}^{-1}\right)}^{-1}$ and Λ=λI. This approximation preserves the upper bound in (18). Consequently, (19) can be reformulated to fit for SOF as

(22) w^k=arg min wkL(w,z˜)=arg min wkλ∑k=1Ky−Φkwk2+2∑l=1Nkz˜k,l12|wk,l|,

which can be solved distributively via the alternating direction method of multipliers (ADMM) algorithm [33] (Section 8.3). The D-R-ARD algorithm for SOF is summarized in Algorithm 1. When using ADMM to solve for ${\hat{w}}_{k}$, the only communication between the agents takes place inside of the ADMM algorithm. The communication load of the ADMM algorithm for SOF is discussed in [33] (Chapter 8).
**Algorithm 1** D-R-ARD for SOF1:${\tilde{z}}_{k}\leftarrow \mathrm{diag}\left\{\Phi_{k}^{T}\Lambda \Phi_{k}\right\}$2:**while** not converged **do**3:    w^←arg min wL(w,z˜)         ▹ See (22); is solved distributively using ADMM [33] (Section 8.3)4:    ${\hat{\gamma }}_{k}\leftarrow \frac{|{\hat{w}}_{k,n}|}{\sqrt{{\tilde{z}}_{k,n}}},\;\forall n=1,\cdots ,N_{k}$5:    ${\tilde{z}}_{k}\leftarrow$ (21)6:$\hat{w}={\left[{\hat{w}}_{1}^{T},\cdots ,{\hat{w}}_{K}^{T}\right]}^{T}$, $\hat{\gamma }={\left[{\hat{\gamma }}_{1}^{T},\cdots ,{\hat{\gamma }}_{K}^{T}\right]}^{T}$

### 2.4. The Distributed Automated Relevance Determination Algorithm for SOE Data Splitting

For SOE, we will assume that measurements y at locations X are partitioned into *K* disjoint subsets ${\left\{y_{k}\left(X_{k}\right),X_{k}\right\}}_{k=1}^{K}$, each associated with the corresponding agent in the network. Hence, each agent *k* makes $M_{k}$ observations $y_{k}\left(X_{k}\right)=\left[y_{k,1}\left(x_{k,1}\right),\ldots ,y_{k,M_{k}}\left(x_{k,M_{k}}\right)\right]$ at locations $X_{k}={\left[x_{k,1},\ldots ,x_{k,M_{k}}\right]}^{T}$, such that $M=\sum_{k=1}^{K}M_{k}$, $y={\left[y_{1}^{T},\ldots ,y_{K}^{T}\right]}^{T}$, $X={\left[X_{1}^{T},\ldots ,X_{K}^{T}\right]}^{T}$, $\Phi ={\left[\Phi_{1}^{T},\ldots ,\Phi_{K}^{T}\right]}^{T}$, and $\eta ={\left[\eta_{1}^{T},\ldots ,\eta_{K}^{T}\right]}^{T}$. This allows us to rewrite (7) in an equivalent form as

(23) $$\begin{array}{c} y=\left[\begin{array}{c} y_{1}\\ \vdots \\ y_{K} \end{array}\right]=\left[\begin{array}{c} \Phi_{1}\\ \vdots \\ \Phi_{K} \end{array}\right]w+\left[\begin{array}{c} \eta_{1}\\ \vdots \\ \eta_{K} \end{array}\right], \end{array}$$

where we assumed that perturbations $\eta_{k}$, k=1,…,K, are independent between agents, i.e.,

$$E\left\{\eta_{k}\eta_{m}^{T}\right\}=\left\{\begin{array}{cc} 0I & k\neq m\\ \lambda_{k}^{-1}I & k=m. \end{array}\right.$$

To cast R-ARD in a distributed setting, we need to be able to solve (19) and compute $\hat{z}$ in (15) over a network of agents. To this end, let us define

(24) $$\begin{array}{cc} D & ≜\Phi^{T}\Lambda \Phi =\sum_{k=1}^{K}\Phi_{k}^{T}\lambda_{k}\Phi_{k}=K\times \underset{\mathrm{averaged}\;\mathrm{consensus}}{\underset{︸}{\frac{1}{K}\sum_{k=1}^{K}\Phi_{k}^{T}\lambda_{k}\Phi_{k}}}. \end{array}$$

where $\Lambda =\mathrm{diag}\left[\lambda_{1}I_{1},\ldots ,\lambda_{K}I_{K}\right]$, and $I_{k}$ is an identity matrix of size $M_{k}\times M_{k}$, k=1,…,K. We point out that D, or rather the last factor in (24), can be computed over a network of agents using an averaged consensus algorithm [34,35].

Next, we apply the Woodbury identity to $\Sigma^{-1}$ to obtain

(25) $$\begin{array}{c} \Sigma^{-1}={\left(\Lambda^{-1}+\Phi \Gamma \Phi^{T}\right)}^{-1}=\Lambda -\Lambda \Phi \Sigma_{w}\Phi^{T}\Lambda , \end{array}$$

where $\Sigma_{w}={\left(\Phi^{T}\Lambda \Phi +\Gamma^{-1}\right)}^{-1}$. Inserting (25) and (24) into (15), we get

(26) $$\begin{array}{c} \hat{z}=\mathrm{diag}\left\{\Phi^{T}\Lambda \Phi -\Phi^{T}\Lambda \Phi \Sigma_{w}\Phi^{T}\Lambda \Phi \right\}=\mathrm{diag}\left\{D-D\Sigma_{w}D\right\}, \end{array}$$

where $\Sigma_{w}={\left(D+\Gamma^{-1}\right)}^{-1}$. Thus, once D becomes available, $\hat{z}$ can be found distributively using expression (26).

To solve (19) distributively, we first note that for the model (23) the likelihood (8) can be equivalently rewritten as

(27) $$p\left(y|w\right)\propto \exp\left\{-\frac{1}{2}\sum_{k=1}^{K}\lambda_{k}{∥y_{k}-\Phi_{k}w∥}^{2}\right\}.$$

It is then straightforward to show that the upper bound (18) will take the form

(28) L(w,γ,z^)≜12∑k=1Kλk∥yk−Φkw∥2+∑l=1Mz^lγl+wl2γl≥L(γ,z^).

Similarly to (18), for any $w_{l}$, l=1,…,M, the bound is minimized with respect to $\gamma_{l}$ at γl=|wl|/z^l, l=1,…,M. Inserting the latter in (28), we obtain an objective function for estimating $w_{l}$

(29) w^=arg min w12∑k=1KλkΦkw−yk22+2∑l=1Mz^l|wl|.

Expression (29) can be readily solved distributively using an ADMM algorithm (see e.g., [33] (Chapter 8) and [36]). Once $\hat{w}$ is found, optimal parameter values $\hat{\gamma }$ are found as γ^l=z^l−12|w^l|, l=1,…,N.

In Algorithm 2, we now summarize the key steps of the resulting D-R-ARD algorithm for SOE. As we can see from Algorithm 2, D-R-ARD includes two optimizing loops. The inner optimization loop is an ADMM algorithm, which is guaranteed to converge to a solution [33]. The convergence of the outer loop is basically the convergence of the R-ARD algorithm presented in [26].
**Algorithm 2** D-R-ARD for SOE 1:${\hat{z}}_{n}\leftarrow 1,\;\forall n=1,\cdots ,N$2:Compute D using averaged consensus over $\Phi_{k}^{T}\Lambda \Phi_{k}$ as in (24)3:**while** not converged **do**4:    w^←arg min wL(w,γ,z^) ▹ See (29); is solved distributively using ADMM [33,36]5:    $\hat{\gamma }\leftarrow \frac{|{\hat{w}}_{n}|}{\sqrt{{\hat{z}}_{n}}},\;\forall n=1,\cdots ,N$6:    $\Sigma_{w}\leftarrow {\left(D+\Gamma^{-1}\right)}^{-1}$7:    $\hat{z}\leftarrow$ (26)

#### Communication Load of D-R-ARD

In the D-R-ARD algorithm, two communication steps are required. The first communication step involves the computation of the matrix D, where we leverage an average consensus algorithm. There, each of the A∈N consensus steps requires the transmission of N(N+1)/2 floats due to the symmetry of D. Note that the number *A* of averaged consensus iterations can vary depending on the connectivity of the network.

The second communication step involves the iterative estimation of the model parameters. Assuming that the update loop of D-R-ARD requires I∈N iterations, the distributed estimation of parameters $\hat{w}$ with R∈N ADMM iterations then scales up as O(I×ARN). Thus, the total communication load of D-R-ARD algorithm behaves as AN(N+1)/2+O(I×ARN). Please note also that for this estimation of the communication load, the network structure remains unchanged.

## 3. Distributed Entropy-Driven Exploration for Sparse Bayesian Learning

The learning algorithm described in the previous section estimates the parameters of the model w and γ given the measurements y and X. In the following, we focus on the question of how a new measurement is acquired in an optimal fashion. As we will show, the main criterion for this purpose is the information or, more specifically, the entropy change as a function of a possible sampling location.

### 3.1. D-Optimality

One possible strategy to optimally select a new measurement location $\tilde{x}$ is provided by the theory of optimal experiment design. Optimal experiment design aims at optimizing the variance of an estimator through a number of optimality criteria. One of these criteria is a so-called D-optimality: it measures the “size” of an estimator covariance matrix by computing the volume of the corresponding uncertainty ellipsoid. More specifically, a determinant (or rather the logarithm of a determinant) of the covariance matrix is computed. The latter can then be optimized with respect to the experiment parameter. In our case, the covariance matrix $\Sigma_{w}$ of the model parameters w is readily given in (12) as a second central moment of p(w|y). Thus, the D-optimality criterion can be formulated as

(30) $$\begin{array}{c} \min\log\left|\Sigma_{w}\left(X,\Pi \right)\right|, \end{array}$$

where the dependency of $\Sigma_{w}$ on measurement locations X has been made explicit. Note that due to the normality of the posterior pdf p(w|y), the term $\log\left|\Sigma_{w}\left(X,\Pi \right)\right|$ is proportional to the entropy of w; thus, minimization of the criterion (30) would imply a reduction of the entropy of the parameter estimates. Note that in contrast to [14], the covariance matrix is not approximated here, but it is computed exactly based on the resulting probabilistic inference model. Our intention is now to evaluate and optimize (30) as a function of the new possible sampling location $\tilde{x}$.

Let us consider a modification of the model (7) as a function of the location $\tilde{x}$. The incorporation of $\tilde{x}$ into (7) would imply that the design matrix Φ would be extended as

(31) $$\begin{array}{c} \tilde{\Phi }\left({\left[X^{T},\tilde{x}\right]}^{T},{\left[\Pi^{T},\tilde{\pi }\right]}^{T}\right)=\left[\begin{array}{cc} \Phi (X,\Pi ) & \phi (X,\tilde{\pi })\\ \phi^{T}\left(\tilde{x},\Pi \right) & \phi (\tilde{x},\tilde{\pi }) \end{array}\right], \end{array}$$

where $\tilde{\pi }$ is a new parameterization of a function ϕ based on the new location $\tilde{x}$—a new regression feature. Let us stress that in general, the potential measurement at $\tilde{x}$ does not have to lead to a new column in (31)—columns, i.e., basis functions in Φ can be fixed from the initial design of the problem. In the latter case, Φ would be extended only by a row vector $\phi^{T}\left(\tilde{x},\Pi \right)=\left[\phi \left(\tilde{x},\pi_{1}\right),\cdots ,\phi \left(\tilde{x},\pi_{N}\right)\right]$. However, a basis function with a currently zero parameter weight estimate might be useful for explaining the new measurement value at $\tilde{x}$ and, thus, might be activated. Our next step is to consider how

1. The D-optimality criterion can be evaluated efficiently for the “grown” design matrix $\tilde{\Phi }$ in (31),
2. And how the criterion can be evaluated in a distributed fashion.

#### 3.1.1. Measurement Only-Update of the D-Optimality Criterion

We will begin with considering the update of the D-optimality criterion with respect to a new measurement location $\tilde{x}$ assuming that only the number of rows in Φ grows, while the number of features stays constant. In this case, (31) can be represented as

(32) $$\begin{array}{c} \tilde{\Phi }\left({\left[X^{T},\tilde{x}\right]}^{T},\Pi \right)=\left[\begin{array}{c} \Phi (X,\Pi )\\ \phi^{T}\left(\tilde{x},\Pi \right) \end{array}\right]. \end{array}$$

Based on (32), the new covariance matrix ${\tilde{\Sigma }}_{w}$ that accounts for the new measurement location $\tilde{x}$ can be computed as

(33) $$\begin{array}{c} {\tilde{\Sigma }}_{w}\left(X,\Pi ,\tilde{x}\right)={\left(\tilde{\Phi }\left({\left[X^{T},\tilde{x}\right]}^{T},\Pi \right)\tilde{\Lambda }\tilde{\Phi }\left({\left[X^{T},\tilde{x}\right]}^{T},\Pi \right)+{\hat{\Gamma }}^{-1}\right)}^{-1}, \end{array}$$

where $\tilde{\Lambda }=\mathrm{diag}\left\{\Lambda ,\overset{˜}{\lambda }\right\}\in R^{M+1\times M+1}$ and $\overset{˜}{\lambda }$ is the assumed noise precision at the potential measurement location. It is worth noting that we assume every measurement to be independent white Gaussian noise.

By combining terms that depend on $\tilde{x}$, we can represent (33) as

(34) $$\begin{array}{cc} {\tilde{\Sigma }}_{w}{\left(X,\Pi ,\tilde{x}\right)}^{-1}= & \left[\begin{array}{c} \Phi^{T}\Lambda \Phi +{\hat{\Gamma }}^{-1} \end{array}\right]+\overset{˜}{\lambda }\phi \left(\tilde{x},\Pi \right)\phi {\left(\tilde{x},\Pi \right)}^{T}\\ = & \Sigma_{w}^{-1}+\overset{˜}{\lambda }\phi \left(\tilde{x},\Pi \right)\phi {\left(\tilde{x},\Pi \right)}^{T}. \end{array}$$

As we see from (34), an addition of a new measurement row causes a rank-1 perturbation of the information matrix $\Sigma_{w}^{-1}$. Using matrix determinant lemma [37], we can thus compute

(35) $$\begin{array}{cc} \log|{\tilde{\Sigma }}_{w}\left(X,\Pi ,\tilde{x}\right)|= & -\log|\Sigma_{w}^{-1}+\overset{˜}{\lambda }\phi \left(\tilde{x},\Pi \right)\phi {\left(\tilde{x},\Pi \right)}^{T}| \end{array}$$

(36) $$\begin{array}{cc} = & \log|\Sigma_{w}|-\log\left|1+\overset{˜}{\lambda }\phi {\left(\tilde{x},\Pi \right)}^{T}\Sigma_{w}\phi \left(\tilde{x},\Pi \right)\right| \end{array}$$

Note that $\Sigma_{w}$ is independent of $\tilde{x}$, and thus, only the second term on the right-hand side of (36) is relevant for the estimation.

Finally, the D-optimality criterion with respect to a location $\tilde{x}$ can be formulated as

(37) arg min x˜log|Σ˜w|≡arg max x˜log1+λ˜ϕ(x˜,Π)TΣwϕ(x˜,Π)=arg max x˜logf(x˜,λ˜),

where we have exchanged minimization with a maximization by changing the sign of the cost function.

#### 3.1.2. Computation of the D-Optimality Criterion with Addition of a New Feature

The computation of the D-optimality criterion becomes more involved when a measurement at a location $\tilde{x}$ is associated with a new feature $\tilde{\pi }$. This can happen if, e.g., $\tilde{\pi }$ is a center or location of a new basis function.

Then, based on (31), the new covariance matrix ${\tilde{\Sigma }}_{w}$ that accounts for $\tilde{x}$ and $\tilde{\pi }$ is formulated as

(38) $$\begin{array}{c} {\tilde{\Sigma }}_{w}\left(X,\Pi ,\tilde{x},\tilde{\pi }\right)={\left({\tilde{\Phi }}^{T}\left({\left[X^{T},\tilde{x}\right]}^{T},{\left[\Pi^{T},\tilde{\pi }\right]}^{T}\right)\tilde{\Lambda }\tilde{\Phi }\left({\left[X^{T},\tilde{x}\right]}^{T},{\left[\Pi^{T},\tilde{\pi }\right]}^{T}\right)+\left[\begin{array}{cc} {\hat{\Gamma }}^{-1} & 0\\ 0 & {\overset{˜}{\gamma }}^{-1} \end{array}\right]\right)}^{-1}, \end{array}$$

where $\overset{˜}{\gamma }$ is a sparsity parameter associated with a new column ${\left[\phi^{T}\left(X,\tilde{\pi }\right),\phi \left(\tilde{x},\tilde{\pi }\right)\right]}^{T}$. By combining terms that depend on $\tilde{x}$, we can represent (38) as

(39) $$\begin{array}{cc} {\tilde{\Sigma }}_{w}{\left(X,\Pi ,\tilde{x},\tilde{\pi }\right)}^{-1}=\; & \left[\begin{array}{cc} \Phi^{T}\Lambda \Phi +{\hat{\Gamma }}^{-1} & \Phi^{T}\Lambda \phi \left(X,\tilde{\pi }\right)\\ \phi^{T}\left(X,\tilde{\pi }\right)\Lambda \Phi & \phi^{T}\left(X,\tilde{\pi }\right)\Lambda \phi \left(X,\tilde{\pi }\right)+{\overset{˜}{\gamma }}^{-1} \end{array}\right]\\ \; & +\overset{˜}{\lambda }\left[\begin{array}{c} \phi (\tilde{x},\Pi )\\ \phi (\tilde{x},\tilde{\pi }) \end{array}\right]{\left[\begin{array}{c} \phi (\tilde{x},\Pi )\\ \phi (\tilde{x},\tilde{\pi }) \end{array}\right]}^{T}. \end{array}$$

To simplify the notation, let us define

(40) $$\begin{array}{c} c\left(\tilde{\pi }\right)≜\Phi^{T}\Lambda \phi \left(X,\tilde{\pi }\right),\;b\left(\tilde{\pi }\right)≜\phi^{T}\left(X,\tilde{\pi }\right)\Lambda \phi \left(X,\tilde{\pi }\right)+{\overset{˜}{\gamma }}^{-1}, \end{array}$$

which can be inserted into (39), leading to

(41) $$\begin{array}{c} {\tilde{\Sigma }}_{w}{\left(X,\Pi ,\tilde{x},\tilde{\pi }\right)}^{-1}=\left[\begin{array}{cc} \Sigma_{w}^{-1} & c(\tilde{\pi })\\ c^{T}\left(\tilde{\pi }\right) & b(\tilde{\pi }) \end{array}\right]+\overset{˜}{\lambda }\left[\begin{array}{c} \phi (\tilde{x},\Pi )\\ \phi (\tilde{x},\tilde{\pi }) \end{array}\right]\left[\begin{array}{cc} \phi^{T}\left(\tilde{x},\Pi \right) & \phi (\tilde{x},\tilde{\pi }) \end{array}\right]. \end{array}$$

The first term in (41) describes how much the new feature column contributes to the covariance matrix, while the second term represents the contribution of a measurement at location $\tilde{x}$. Let us now insert (41) into the D-optimality criterion in (30). By applying the matrix determinant lemma [37] to the resulting expression, we compute

(42) $$\begin{array}{cc} \log|{\tilde{\Sigma }}_{w}\left(X,\Pi_{N},\tilde{x},\tilde{\pi }\right)|= & -\log\left|\begin{array}{cc} \Sigma_{w}^{-1} & c(\tilde{\pi })\\ c{\left(\tilde{\pi }\right)}^{T} & b(\tilde{\pi }) \end{array}\right|\\ - & \log\left|\begin{array}{c} 1+\overset{˜}{\lambda }{\left[\begin{array}{c} \phi (\tilde{x},\Pi )\\ \phi (\tilde{x},\tilde{\pi }) \end{array}\right]}^{T}{\left[\begin{array}{cc} \Sigma_{w}^{-1} & c(\tilde{\pi })\\ c{\left(\tilde{\pi }\right)}^{T} & b(\tilde{\pi }) \end{array}\right]}^{-1}\left[\begin{array}{c} \phi (\tilde{x},\Pi )\\ \phi (\tilde{x},\tilde{\pi }) \end{array}\right] \end{array}\right|. \end{array}$$

Now, consider separately the contribution of the two terms in the right-hand side of (42) to the D-optimality criterion. For the first term, we can use the Schur complement [38] $q\left(\tilde{\pi }\right)=b\left(\tilde{\pi }\right)-c^{T}\left(\tilde{\pi }\right)\Sigma_{w}c\left(\tilde{\pi }\right)$ such that the first logarithmic term can be reformulated as

(43) $$\begin{array}{c} \log\left|\begin{array}{cc} \Sigma_{w}^{-1} & c(\tilde{\pi })\\ c{\left(\tilde{\pi }\right)}^{T} & b(\tilde{\pi }) \end{array}\right|=-\log\left|\Sigma_{w}\right|+\log q\left(\tilde{\pi }\right). \end{array}$$

Note that $\Sigma_{w}$ is independent of $\tilde{x}$ and of $\tilde{\pi }$, which is a fact that will become useful later.

To simplify the second term in the right-hand side of (42), we first apply inversion rules for structured matrices [39], which allows us to write

(44) $$\begin{array}{c} {\left[\begin{array}{cc} \Sigma_{w}^{-1} & c(\tilde{\pi })\\ c{\left(\tilde{\pi }\right)}^{T} & b(\tilde{\pi }) \end{array}\right]}^{-1}=\left[\begin{array}{cc} \Sigma_{w}-\Sigma_{w}c\left(\tilde{\pi }\right)q{\left(\tilde{\pi }\right)}^{-1}c{\left(\tilde{\pi }\right)}^{T}\Sigma_{w} & -\Sigma_{w}c\left(\tilde{\pi }\right)/q\left(\tilde{\pi }\right)\\ -c{\left(\tilde{\pi }\right)}^{T}\Sigma_{w}/q\left(\tilde{\pi }\right) & 1/q(\tilde{\pi }), \end{array}\right] \end{array}$$

and thus

(45) $$\begin{array}{cc} \; & \log\left|\begin{array}{c} 1+{\left[\begin{array}{c} \phi (\tilde{x},\Pi )\\ \phi (\tilde{x},\tilde{\pi }) \end{array}\right]}^{T}{\left[\begin{array}{cc} \Sigma_{w}^{-1} & c(\tilde{\pi })\\ c{\left(\tilde{\pi }\right)}^{T} & b(\tilde{\pi }) \end{array}\right]}^{-1}\left[\begin{array}{c} \phi (\tilde{x},\Pi )\\ \phi (\tilde{x},\tilde{\pi }) \end{array}\right] \end{array}\right|\\ \; & =\log\left(1+\overset{˜}{\lambda }\phi^{T}\left(\tilde{x},\Pi \right)\Sigma_{w}\phi \left(\tilde{x},\Pi \right)+\overset{˜}{\lambda }{\left(\phi \left(\tilde{x},\tilde{\pi }\right)-c{\left(\tilde{\pi }\right)}^{T}\Sigma_{w}\phi \left(\tilde{x},\Pi \right)\right)}^{2}/q\left(\tilde{\pi }\right)\right)\\ \; & =\log\left(f(\tilde{x},\overset{˜}{\lambda })+\overset{˜}{\lambda }{\left(\phi \left(\tilde{x},\tilde{\pi }\right)-c{\left(\tilde{\pi }\right)}^{T}\Sigma_{w}\phi \left(\tilde{x},\Pi \right)\right)}^{2}/q\left(\tilde{\pi }\right)\right). \end{array}$$

Finally, after inserting (43) and (45) into (42), the D-optimality criterion with respect to a location $\tilde{x}$ can be formulated as

(46) arg min x˜log|Σ˜w(X,Π,x˜,π˜)|≡arg max x˜logq(π˜)f(x˜,λ˜)+λ˜ϕ(x˜,π˜)−c(π˜)TΣwϕ(x˜,Π)2,

where we have exchanged minimization with a maximization by changing the sign of the cost function, and we dropped $\log|\Sigma_{w}|$ as it is independent of $\tilde{x}$ and $\tilde{\pi }$.

#### 3.1.3. Distributed Computation of the D-Optimality Criterion for SOE

Let us begin first with evaluating the D-optimality criterion for the SOE case. Evaluating (37) for this data splitting is easier as compared with SOF.

Since Π is known to each agent, the vector $\phi (\tilde{x},\Pi )$ can be evaluated without any cooperation between the agents. The covariance $\Sigma_{w}$ can then be evaluated distributively using averaged consensus as $\Sigma_{w}={\left(D+{\hat{\Gamma }}^{-1}\right)}^{-1}$, where D is computed using network-wide averaging. To compute (46), a few more steps are needed. Specifically, in addition to $\Sigma_{w}$, we also need to compute the quantities $c(\tilde{\pi })$ and $b(\tilde{\pi })$ in (40) to evaluate the criterion. These can already be computed using averaged consensus as

(47) $$\begin{array}{cc} c(\tilde{\pi }) & =\Phi^{T}\Lambda \phi \left(X,\tilde{\pi }\right)=K\times \frac{1}{K}\sum_{k=1}^{K}\Phi_{k}^{T}\Lambda \phi \left(X_{k},\tilde{\pi }\right), \end{array}$$

(48) $$\begin{array}{cc} b(\tilde{\pi }) & =\phi {\left(X,\tilde{\pi }\right)}^{T}\Lambda \phi \left(X,\tilde{\pi }\right)+{\tilde{\gamma }}^{-1}=K\times \frac{1}{K}\sum_{k=1}^{K}\phi {\left(X_{k},\tilde{\pi }\right)}^{T}\Lambda \phi \left(X_{k},\tilde{\pi }\right)+{\overset{˜}{\gamma }}^{-1}. \end{array}$$

Then, using (47) and (48) as well as $\Sigma_{w}$ computed distributively, the criterion (46) can be easily evaluated by each agent.

It is worth noting that the choice of ${\overset{˜}{\gamma }}^{-1}$ in (48) is the only parameter that can be set manually in this exploration criterion. Basically, it controls how much we know about the potential measurement location. If ${\overset{˜}{\gamma }}^{-1}$ is large, the criterion would yield that the potential measurement location is not informative. On the other side, if ${\overset{˜}{\gamma }}^{-1}\to 0$, the criterion yields that the considered measurement location is potentially informative. We set ${\overset{˜}{\gamma }}^{-1}=0$ for all considered measurement locations, such that the current information in the model determines how informative a measurement location could be.

#### 3.1.4. Distributed Computation of the D-Optimality Criterion for SOF

For SOF, (37) is unsuited for a distributed computation such that some changes have to be made. First, we define the following terms to facilitate the distributed formulation

(49) $$\begin{array}{cc} H & ≜\Phi \hat{\Gamma }\Phi^{T}=K\times \frac{1}{K}\sum_{k=1}^{K}\Phi_{k}{\hat{\Gamma }}_{k}\Phi_{k}^{T}, \end{array}$$

(50) $$\begin{array}{cc} d & ≜\Phi \hat{\Gamma }\phi \left(\tilde{x},\Pi \right)=K\times \frac{1}{K}\sum_{k=1}^{K}\Phi_{k}{\hat{\Gamma }}_{k}\phi \left(\tilde{x},\Pi_{k}\right), \end{array}$$

(51) $$\begin{array}{cc} v & ≜\phi^{T}\left(\tilde{x},\Pi \right)\hat{\Gamma }\phi \left(\tilde{x},\Pi \right)=K\times \frac{1}{K}\sum_{k=1}^{K}\phi_{k}^{T}\left(\tilde{x},\Pi_{k}\right){\hat{\Gamma }}_{k}\phi \left(\tilde{x},\Pi_{k}\right), \end{array}$$

where $\Pi_{k}={\left[\pi_{1},\cdots ,\pi_{N_{k}}\right]}^{T}\in R^{N_{k}\times s}$ and ${\hat{\Gamma }}_{k}={\left[{\hat{\gamma }}_{1},\cdots ,{\hat{\gamma }}_{N_{k}}\right]}^{T}$. All terms in (49)–(51) can then be computed by means of an averaged consensus [40,41]. Next, we reformulate $\Sigma_{w}$ with the help of the matrix-inversion-lemma as

(52) $$\begin{array}{c} \Sigma_{w}=\hat{\Gamma }-\hat{\Gamma }\Phi^{T}{\left(\Lambda^{-1}+\Phi \hat{\Gamma }\Phi^{T}\right)}^{-1}\Phi \hat{\Gamma }=\hat{\Gamma }-\hat{\Gamma }\Phi^{T}{\left(\Lambda^{-1}+H\right)}^{-1}\Phi \hat{\Gamma }. \end{array}$$

Now, (37) can be reformulated in a distributed setting for SOF as

(53) $$\begin{array}{cc} f(\tilde{x},\overset{˜}{\lambda }) & =1+\overset{˜}{\lambda }\phi^{T}\left(\tilde{x},\Pi \right)\Sigma_{w}\phi \left(\tilde{x},\Pi \right)\\ \; & =1+\overset{˜}{\lambda }\phi^{T}\left(\tilde{x},\Pi \right)\left(\hat{\Gamma }-\hat{\Gamma }\Phi^{T}{\left(\Lambda^{-1}+H\right)}^{-1}\Phi \hat{\Gamma }\right)\phi \left(\tilde{x},\Pi \right)\\ \; & =1+\overset{˜}{\lambda }\phi^{T}\left(\tilde{x},\Pi \right)\hat{\Gamma }\phi \left(\tilde{x},\Pi \right)-\overset{˜}{\lambda }\phi^{T}\left(\tilde{x},\Pi \right)\hat{\Gamma }\Phi^{T}{\left(\Lambda^{-1}+H\right)}^{-1}\Phi \hat{\Gamma }\phi \left(\tilde{x},\Pi \right)\\ \; & =1+\overset{˜}{\lambda }v-\overset{˜}{\lambda }d^{T}{\left(\Lambda^{-1}+H\right)}^{-1}d. \end{array}$$

For the case when the criterion (46) is used for evaluaton of the D-optimality, the variable $q(\tilde{\pi })$ in (46) and the second additive term there have to be reformulated in a form suitable for SOF data splitting. For the former, we utilize the definitions in (49)–(51), together with (52) such that

(54) $$\begin{array}{cc} q(\tilde{\pi }) & =\gamma^{-1}+\phi^{T}\left(X,\tilde{\pi }\right)\Lambda \phi \left(X,\tilde{\pi }\right)-\phi^{T}\left(X,\tilde{\pi }\right)\Lambda \Phi \Sigma_{w}\Phi^{T}\Lambda \phi \left(X,\tilde{\pi }\right)\\ & =\gamma^{-1}+\phi^{T}\left(X,\tilde{\pi }\right)\Lambda \phi \left(X,\tilde{\pi }\right)-\phi^{T}\left(X,\tilde{\pi }\right)\Lambda \left(H-H{\left(\Lambda^{-1}+H\right)}^{-1}H\right)\Lambda \phi \left(X,\tilde{\pi }\right)\\ & =\gamma^{-1}+\phi^{T}\left(X,\tilde{\pi }\right)\Lambda \phi \left(X,\tilde{\pi }\right)-\phi^{T}\left(X,\tilde{\pi }\right)\Lambda {\left(\Lambda +H^{-1}\right)}^{-1}\Lambda \phi \left(X,\tilde{\pi }\right)\\ & =\gamma^{-1}+\phi^{T}\left(X,\tilde{\pi }\right)\left(\Lambda -\Lambda {\left(\Lambda +H^{-1}\right)}^{-1}\Lambda \right)\Lambda \phi \left(X,\tilde{\pi }\right)\phi \left(X,\tilde{\pi }\right)\\ & =\gamma^{-1}+\phi^{T}\left(X,\tilde{\pi }\right){\left(\Lambda^{-1}+H\right)}^{-1}\phi \left(X,\tilde{\pi }\right). \end{array}$$

The other term in (46) is then reformulated similarly using the results (49)–(52) as

(55) $$\begin{array}{cc} c^{T}\left(\tilde{\pi }\right)\Sigma_{w}\phi \left(\tilde{x},\Pi \right) & =\phi^{T}\left(X,\tilde{\pi }\right)\Lambda \Phi \left(\hat{\Gamma }-\hat{\Gamma }\Phi^{T}{\left(\Lambda^{-1}+H\right)}^{-1}\Phi \hat{\Gamma }\right)\phi \left(\tilde{x},\Pi \right)\\ \; & =\phi^{T}\left(X,\tilde{\pi }\right)\Lambda \left(\Phi \hat{\Gamma }\phi \left(\tilde{x},\Pi \right)-\Phi \hat{\Gamma }\Phi^{T}{\left(\Lambda^{-1}+H\right)}^{-1}\Phi \hat{\Gamma }\phi \left(\tilde{x},\Pi \right)\right)\\ \; & =\phi^{T}\left(X,\tilde{\pi }\right)\Lambda \left(d-H{\left(\Lambda^{-1}+H\right)}^{-1}\right)d)\\ \; & =\phi^{T}\left(X,\tilde{\pi }\right)\Lambda \left(I-H{\left(\Lambda^{-1}+H\right)}^{-1}\right)d. \end{array}$$

As a result, the exploration criterion can be re-formulated for SOF in the following form

(56) arg min x˜log|Σ˜w(X,Π,x˜,π˜)|≡arg max x˜logq(π˜)f(x˜,λ˜)+λ˜ϕ(x˜,π˜)−ϕT(X,π˜)Λ(I−H(Λ−1+H)−1)d2,

with $q(\tilde{\pi })$ defined in (54) and $f(\tilde{x},\overset{˜}{\lambda })$ given in (53).

## 4. Experimental Setup

This section describes definition of the experimental setup, calibration of the sensors, and collection of ground-truth data for performance evaluation.

### 4.1. Map Construction

**Figure 1:**
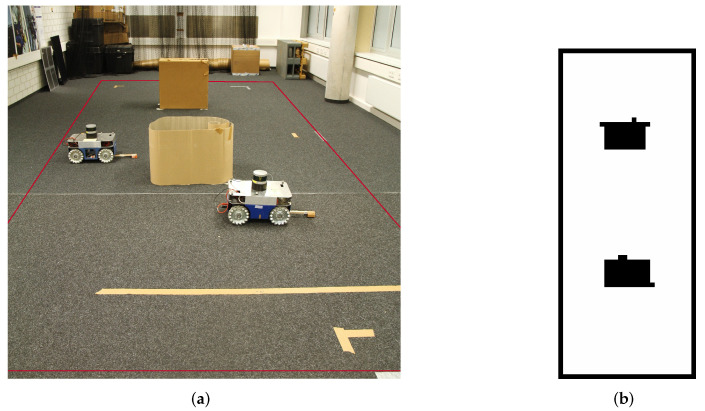
(**a**) The experimental setting with obstacles. The red line indicates the experimental area, where the slider can navigate. (**b**) The constructed map.

The following describes our experimental setup. We conducted the experiments indoor in our laboratory with two paper boxes as obstacles displayed in Figure 1a. Red lines in the figure represent the borders of the experimental area. We use two Commonplace Robotics (https://cpr-robots.com, accessed on 19 March 2022) ground-based robots with mecanum wheels; further in the text, we will refer to the robots as sliders due to their ability to move holonomically. To position the slider within the environment, the laboratory is equipped with 16 VICON (https://www.vicon.com/, accessed on 19 March 2022) Bonita cameras. For the experiment itself, we assume that the map is a priori known to the system. Thus, we need to record the map before the experiment. So, a single slider is equipped with a light detection and ranging (LIDAR) sensor. We use a Velodyne (https://velodynelidar.com/, accessed on 19 March 2022) *VLP-16* LIDAR and the corresponding robot operating system (ROS) package, which can be downloaded from the ROS repository. We construct the map while sending waypoints to the slider manually. The steering of the slider is done with the help of ROS’ *navigation stack* [42] together with the *Teb Local Planner* [43]. The sensor output of the LIDAR and the slider position estimated by the VICON system are then used to generate a map with the *Octomap* [44] ROS package. Because we use the VICON position of the slider, which is accurate, this mapping procedure is simpler compared to simultaneous localization and mapping (SLAM) algorithms [45,46]. Figure 1b shows the constructed map, which is afterwards used in the experiment.

### 4.2. Sensor Calibration

Each slider is equipped with a XSens MTw inertial measurement unit (IMU). The sensor comprises a three-axis magneto-resistive magnetometer, an accelerometer, gyroscopes, and a barometer. For the following experiment, we only use the magnetometer. The sensor is attached to a wooden stick to reduce the influence of the metal wheels on the measurement. Although the sliders are equipped with sensors from the same product line of the same manufacturer, their absolute perception differs. Additionally, the sensors can still perceive the metal in the wheels of the robots. Therefore, we need to calibrate the sensors relatively to each other to perceive the environment equally using the approach proposed in [47].

**Figure 2:**
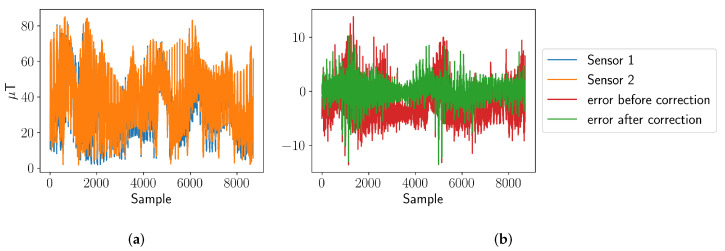
(**a**) Absolute values of the magnetic field samples of two sensors. It is assumed that each sensor measured at the same locations. (**b**) Error of the absolute values of the magnetic field samples before and after the corrections. The calibrated sensor has now the same mean as the reference sensor, and the standard deviation of the error is reduced.

The authors in [47] assume that the sensor readings of one sensor can be expressed as another sensor’s reading through an affine transformation. To estimate the rotation and translation, multiple sensor readings of all sensors have to be acquired. These readings are then exploited to estimate the rotation and translation relative to one specific sensor by means of a least squares method. In this experiment, each magnetic field sensor reads at a position $x_{m}$ one measurement of the magnetic field per Euclidean axis. During the estimation, absolute values of these measurements are used. Figure 2a shows the absolute values of the sensor readings for multiple measurement locations of two sensors. The error of the sensor readings before and after calibration are presented in Figure 2b. The correction thus reduces the bias and the standard deviation of the error between both sensors.

However, this calibration is only useful if the orientation of both sensors is constant during the experiment. As the sensors always measure in the same orientation, this assumption is fulfilled for our experiments. For further information on intrinsic calibration of inertial and magnetic sensors, the reader is referred to [48].

### 4.3. Collecting Ground Truth Data

**Figure 3:**
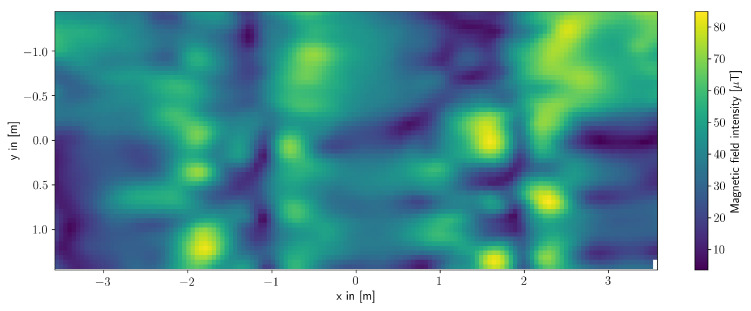
Magnetic field intensity of the Holodeck collected for the experiment with real sensors. The measurements were made in 5 cm steps.

In order to evaluate the performance of the distributed exploration, we also need to know the actual magnetic field in the laboratory—a ground truth data. For collecting the ground truth data, one slider measures the area of the Holodeck in a systematic fashion, where the distance between each measurement was set to be 5 cm such that in total, 8699 measurement points were collected. On each measurement position, multiple sensor readings are taken and averaged. The resulting ground truth is displayed in Figure 3.

## 5. Experimental System Design

Our setup relies on ROS (https://www.ros.org/, accessed on 19 March 2022), which manages the communication between all software modules called *nodes*. On each slider, several ROS nodes are running such as the motor controller, which translates the measurement locations into velocity commands for each wheel, the path-planner, and the sensor.

As a path-planner, we use the popular A* [49,50]. We implemented the A* algorithm as a global and as a local planner, which is utilized for collision avoidance. Therefore, each slider does not only consider the global map but also a local map around its current position.

After receiving a new waypoint, the global path planner estimates a path in the global map from the current position to the goal avoiding the obstacles. If there is no other robotic system in its path, the goal is reached. However, if another slider enters the local frame while the robot is on its way toward the goal, the robot stops, and the path within the local frame is re-planned to avoid collisions. If the planner is not able to find a solution in the local frame within a given time, the global path planning is re-initiated, taking the current slider as an obstacle into account.

**Figure 4:**
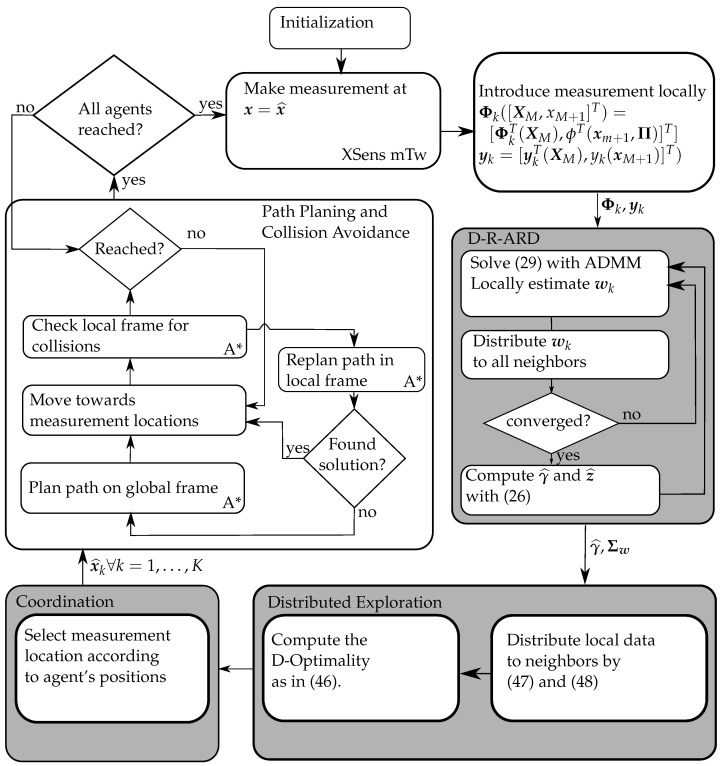
System design with additional path planner and map constraints. Each gray box represents interaction between other agents. In some boxes, the lower right indicates where this process belongs. This software setup is representative for the SOE distribution paradigm.

The whole system design for this experiment is shown in Figure 4. The distributed exploration criterion uses the computed map excluding the locations of the obstacles. In addition, the map information is used by the path-planner to find an obstacle-free path to the estimated measurement location $\hat{x}$. Figure 4 also describes the process-flow of the whole system.

For comparison, we will use non-Bayesian SOF and SOE formulations as discussed in [14]. As in these formulations, the ADMM algorithm [33] was used for estimation, we will refer to them as ADMM for SOF and ADMM for SOE, respectively. For the Bayesian learning and algorithms discussed in this paper, we will refer to them as D-R-ARD for SOF and the D-R-ARD for SOE (see also Table 1).

In the experiments, we will set the number of basis functions to N=560, which also determines the size of the vector w. The basis functions are distributed in a regular grid. We consider Gaussian basis functions with a width set to $\sigma_{n}=0.25$ such that

(57) $$\begin{array}{c} \phi_{n}\left(x,\pi_{n}\right)=\exp\left\{-\frac{∥x-\pi_{n}{∥}^{2}}{2\sigma_{n}^{2}}\right\}, \end{array}$$

where $\pi_{n}\in R^{s}$ and s=d.

After initialization of the system, every agent takes a first measurement and incorporates it in its local measurement model to calculate the first estimate of the regression. Then, each algorithm requires that the intermediate estimated parameter weights are distributed to the neighbors (following Figure 4) to do an average consensus [40,41]. Consequently, each agent can proceed to estimate with the regression using the averaged intermediate parameter weights. When the distributed regression converged, the agents use the estimated covariance matrix in the distributed exploration step. In this step, the agents propose candidate positions to their neighbors and receive information to compute the D-optimality criterion locally. When the best next measurement locations are chosen, they are passed to the coordination part [51] to verify that all agents go to different positions. If the measurement location is considered as valid, an agent locally plans its path on the global frame to reach the goal. While approaching the goal, the agent checks if other agents entered into the local frame to avoid collisions. When all agents reached their goal, the agents take measurements and the process flow continues.

As evaluation metric, we chose the normalized mean square error (NMSE), which can be defined as

(58) $$\begin{array}{c} e≜\frac{∥y_{\mathrm{true}}\left(X_{T}\right)-\Phi \left(X_{T},\Pi \right)\hat{w}∥}{∥y_{\mathrm{true}}\left(X_{T}\right)∥}, \end{array}$$

where $y_{\mathrm{true}}\left(X_{T}\right)\in R^{T}$ is the ground truth measured at T∈N positions $X_{T}\in R^{T\times d}$. Here, we set T=560, and these locations are equal to the center positions of the Gaussian basis functions.

## 6. Experimental Validation

**Figure 5:**
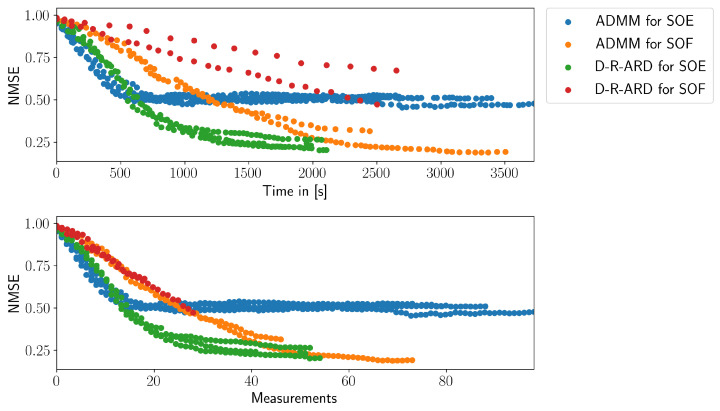
The NMSE of the conducted experiments with respect to time and with respect to the number of measurements.

Figure 5 shows the NMSE of all conducted experiments with respect to time (top plot) and to the number of measurements (bottom plot). Each experimental run has a different duration, and the ROS system uses asynchronous interprocess communication resulting in asynchronous time-steps. Thus, all runs of one particular algorithm are visualized as a scatter plot. The number of measurements varies because the computation time for each measurement could be different. As a consequence, an averaging along multiple experimental runs along the time axis is not reasonable. For both ADMM algorithms, we conducted four experiments, whereas for each D-R-ARD algorithm, we conducted two experiments. The corresponding results are summarized in Figure 5.

When looking at the top plot in Figure 5, the D-R-ARD for SOE has the best performance because the NMSE is reduced faster compared to the other methods.

Regarding the ADMM algorithms, the SOE paradigm has a brief benefit until the 1200 s until SOF paradigm outperforms the SOE paradigm. The weak performance of D-R-ARD for SOF might result from the distributed structure of the algorithm, which requires the algorithm to compute a matrix inversion in each iteration together with the computational complex estimation of parameter weights and variances. In contrast to that, the corresponding algorithm with the SOE distribution paradigm is able to cache the matrix inversion, which drastically increases the performance. Yet, the D-R-ARD algorithms have generally a higher computational complexity compared to the ADMM algorithms. This is due to the fact that the Bayesian methods require the covariance to be computed in each iteration. The ADMM algorithm, in contrast, does not require this.

The plot at the bottom of Figure 5 displays the NMSE with respect to the number of obtained measurements. There, the D-R-ARD for SOF and ADMM for SOF have almost the same performance. However, the ADMM for SOF is able to achieve substantially more measurements because it is computationally less complex. Consequently, the ADMM for SOF achieves not only more measurements but is on a par with the D-R-ARD for SOF when it comes to efficiency per measurement.

For the SOE distribution paradigm, on the contrary, it is beneficial to use the Bayesian methodology. In the experiments we present here, the D-R-ARD for SOE achieves a lower NMSE with fewer measurements compared to ADMM for SOE algorithm. This could be due to the fact that D-R-ARD for SOE computes the entropy of the parameter weights and does not approximate it. The computed entropy seems then to be better for the D-optimality criterion than the approximated version for the ADMM for SOE.

**Figure 6:**
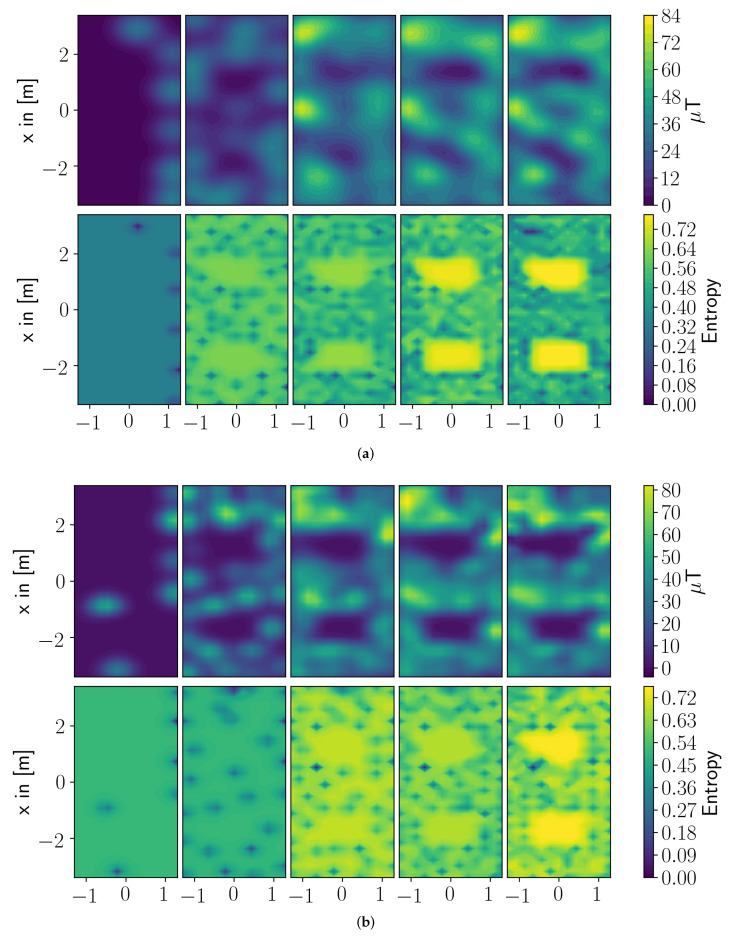
(**a**) SOE with a classic framework. (**b**) SOE with a Bayesian framework. In both figures, the upper row displays the estimates at different time steps and the lower row shows the entropy at the same time steps.

To support the claim that the Bayesian framework estimates a better covariance of the parameter weights when the SOE paradigm is applied, Figure 6a,b present the estimated magnetic field and the estimated covariance at different timesteps. In both figures, the left most plots display the beginning of the experiment and the most right plots show the end result of the experiment. At the beginning of the experiments, both algorithms—ADMM and D-R-ARD for SOE—estimate a sparse covariance with not much difference. As the measurements increase, the approximated covariance becomes smoother, and the covariance estimated in the Bayesian framework stays sparse. This effect might result from the approximation of a covariance as introdcued in [14], where a penalty parameter needs to be chosen as a compromise between sparsity and a reasonably well approximated covariance.

As a second remark, the ADMM algorithms involve a thresholding operator, which sets all not used basis functions to zero such that these basis functions can not be considered by the exploration step. This is controlled by a manually set penalty parameter and might be sub-optimal. The D-R-ARD for SOE, on the other side, estimates a hyper-parameter for each basis function based on the current data. Therefore, the influence of each basis function is addressed more individually and, hence, leads to a better covariance estimate. The way basis functions and parameter weights are introduced in the SOF paradigm makes this effect eventually not observable between the Bayesian and the Frequentist framework.

## 7. Conclusions

The presented paper proposes and validates a method for spatial regression using Sparse Bayesian Learning (SBL) and exploration, which are both implemented over a network of interconnected mobile agents. The spatial process of interest is described as a linear combination of parameterized basis functions; by constraining the weights of these functions in the final representation using a sparsifying prior, we find a model with only a few, relevant functions contributing to the model. The learning is implemented in a distributed fashion, such that no centralized processing unit is necessary. We also considered two conceptually different distribution paradigms splitting-over-features (SOF) and splitting-over-examples (SOE). To this end, a numerical algorithm based on alternating direction method of multipliers is used.

The learned representation is used to devise an information-driven optimal data collection approach. Specifically, the perturbation of the parameter covariance matrix with respect to a new measurement location is derived. This perturbation allows us to choose new measurement locations for agents such that the size of the resulting joint parameter uncertainty, as measured by the log-determinant of the covariance, is minimized. We show also how this criterion can be evaluated in a distributed fashion for both distribution paradigms in an SBL framework.

The resulting scheme thus includes two key steps: (i) cooperative sparse models that fit data collected by agents, and (ii) the cooperative identification of new measurement locations that optimizes the D-optimality criterion. To validate the performance of the scheme, we set up an experiment involving two mobile robots that navigated in an environment with obstacles. The robots were tasked with reconstructing the magnetic field which was measured on the floor of the laboratory by a magnetometer sensor. We tested the proposed scheme against a non-Bayesian sparse regression method and a similar exploration criterion.

The experimental results show that the Bayesian methods explore more efficiently than the benchmark algorithms. Efficiency is measured as the reduction of error over the number of measurements or the reduction of error over time. The reason is that the used Bayesian method directly computes the covariance matrix from the data and has fewer parameters that have to be manually adjusted. The exploration with these methods is therefore simpler to set up as compared with non-Bayesian inference approaches studied before. Yet, for the SOF distribution paradigm, the Bayesian method is computationally too demanding such that significantly fewer measurements can be collected in the same amount of time as compared with the non-Bayesian learning method.

## Figures and Tables

**Table 1:** The algorithms that are used in this experiment and where they are introduced.

	Algorithm Introduced in	Exploration Introduced in
ADMM for SOE	[33]	[14]
ADMM for SOF	[33]	[14]
D-R-ARD for SOE	Section 2.4	Section 3.1.3
D-R-ARD for SOF	[15]	Section 3.1.4

## Abbreviations

The following abbreviations are used in this manuscript:

**ADMM**	alternating direction method of multipliers
**ROS**	robot operating system
**SBL**	Sparse Bayesian Learning
**PDF**	probability density function
**SOF**	splitting-over-features
**SOE**	splitting-over-examples
**NMSE**	normalized mean square error
**D-R-ARD**	distributed R-ARD
**R-ARD**	reformulated automatic relevance determination
**IMU**	inertial measurement unit
**LIDAR**	light detection and ranging
**SLAM**	simultaneous localization and mapping
**UAV**	unmanned aerial vehicle
**LASSO**	least absolute shrinkage and selection operator
**MAP**	maximum a posteriori
